# Microbial Dysbiosis: Rethinking Disease in Marine Ecosystems

**DOI:** 10.3389/fmicb.2016.00991

**Published:** 2016-06-21

**Authors:** Suhelen Egan, Melissa Gardiner

**Affiliations:** Centre for Marine Bio-Innovation, School of Biological, Earth and Environmental Sciences, The University of New South Wales, SydneyNSW, Australia

**Keywords:** dysbiosis, marine diseases, opportunistic pathogens, microbial interactions, microbiome, holobiont

## Abstract

With growing environmental pressures placed on our marine habitats there is concern that the prevalence and severity of diseases affecting marine organisms will increase. Yet relative to terrestrial systems, we know little about the underlying causes of many of these diseases. Moreover, factors such as saprophytic colonizers and a lack of baseline data on healthy individuals make it difficult to accurately assess the role of specific microbial pathogens in disease states. Emerging evidence in the field of medicine suggests that a growing number of human diseases result from a microbiome imbalance (or dysbiosis), questioning the traditional view of a singular pathogenic agent. Here we discuss the possibility that many diseases seen in marine systems are, similarly, the result of microbial dysbiosis and the rise of opportunistic or polymicrobial infections. Thus, understanding and managing disease in the future will require us to also rethink definitions of disease and pathogenesis for marine systems. We suggest that a targeted, multidisciplinary approach that addresses the questions of microbial symbiosis in both healthy and diseased states, and at that the level of the holobiont, will be key to progress in this area.

## Introduction

Almost two decades on, the wealth of microbial biodiversity uncovered by the molecular ecology revolution continues to amaze. Microbiome studies are being reported at a rapid rate, and include ecosystems ranging from the deep oceans to the upper troposphere (DeLeon-Rodriguez et al., 2013; Inagaki et al., 2015). It is now widely accepted that microorganisms have important functions (albeit often not well understood), not only for our personal health, but also for the survival of our planet (Alivisatos et al., 2015).

In the marine environment, microorganisms constitute over 90% of the living biomass where they are essential for nutrient cycling and form intimate associations (symbiosis) with all other marine organisms. Microbial symbioses in marine environments can take many forms, and today one might argue that the excellent textbook cases of a single-symbiont to a single-host organism [e.g., *Euprymna scolopes* and *Vibrio fischeri* (McFall-Ngai, 2014)] are rare. Rather, most interactions between microorganisms and their host/s involve multiple partners that work in multifaceted ways to impact host development and physiology (Wahl et al., 2012), such as in the case for sponges (Webster and Thomas, 2016), corals (Rädecker et al., 2015), and algae (Egan et al., 2013; Abby et al., 2014; Amin et al., 2015).

However microbial–host interactions are not always beneficial and marine organisms are reported to suffer from a variety of disease symptoms, often as a result of a yet unknown etiology (Munn, 2006; Bourne et al., 2009; Gachon et al., 2010; Morado, 2011; Egan et al., 2014). Disease events in the marine environment not only impact directly on the host population, but can also result in ecosystem-wide impacts due to, for example, the mass mortality of keystone species (Burge et al., 2013). These events are predicted to increase with global climate change and elevating anthropogenic pressures (Gattuso et al., 2015). Hence, there is an urgent need to generate data that speak to both the causes and the environmental factors mitigating disease in marine systems. As a guide to understand the complexity of marine diseases, marine scientists may need to look to advances in the human microbiome field. In the past decade, research into human disease has suggested that many chronic diseases (including skin, bowel, and lung disorders) are driven by a disturbance (or shift) in the natural microbiome [i.e., dysbiosis (see **Table 1** for definition)] rather than a singular etiological agent (Althani et al., 2015). Here, we propose that many diseases in marine systems can also be viewed from the point of microbial dysbiosis, analogous to some chronic diseases in humans. However, while a conceptual framework can be gained from studies of dysbiosis in the medical field, the nature of marine systems and the inherent differences between humans and marine organisms may necessitate a specific set of approaches and working models.

**Table 1:** Definition of terminology commonly used to describe microbial–host interactions in the context of host health and disease.

Term	Definition	Further reading
Commensal	A host-associated organism that does not induce host damage upon colonization	Casadevall and Pirofski, 2000
Disease	The health outcome of an organism where normal function is impaired after damage (often induced by a microbe(s)) has occurred.	Casadevall and Pirofski, 2003
Dysbiosis	A microbial community shift that has a negative impact on the host.	Petersen and Round, 2014
Holobiont	A host organism and the entirety of its microbial community, under normal conditions, and in the absence of disease.	Rosenberg et al., 2007
Koch’s postulates	A microorganism-centric methodology used to demonstrate a causal relationship between a pathogen and a disease. The postulates commonly cited are: the pathogen must be present in each case of the disease and absent from healthy individuals; and when isolated in pure culture and used to experimentally infect an individual, the pathogen must induce the disease.	Kaufmann and Schaible, 2005
Opportunistic pathogen	An organism that is capable of causing damage to a host under specific conditions, but may also exist as a commensal on the same host.	Casadevall and Pirofski, 2000
Polymicrobial infection	Disease as a result of the co-infection by multiple microorganisms	Hajishengallis and Lamont, 2016
Saprophyte	An organism that lives and proliferates on dead or already diseased hosts. Often a secondary invader or opportunist.	Willey et al., 2008
Virulence	A relative measure of a microorganism’s ability to induce disease on a host.	Méthot and Alizon, 2014
‘Dual role’ virulence factor	A microbial trait (molecule, protein, etc.) that has direct/indirect roles in both environmental survival/persistence and host disease progression.	Vezzulli et al., 2008

## Are Emerging Diseases in Marine Systems Related to Dysbiosis and Opportunistic Pathogens?

The last decade has seen an increase in the reporting of disease syndromes of cultured and natural populations of marine organisms including fish, seagrass, seaweeds, corals, and other invertebrates (Harvell et al., 2002, 2004; Lafferty et al., 2004; Ward and Lafferty, 2004; Bourne et al., 2009; Gachon et al., 2010). However, in many cases the causative agent/s of the disease are unknown; and indeed debate over the importance of microbial pathogens in relatively well-studied marine diseases continues (Hoegh-Guldberg et al., 2007; Rosenberg et al., 2009; Gachon et al., 2010; Burge et al., 2013).

The first likely explanation for the uncertainty over the microbial involvement in particular marine diseases is due to the difficulty in distinguishing pathogens from other saprophytic bacteria that proliferate on a diseased or decaying host (Burge et al., 2013; Egan et al., 2014). Adding to this uncertainty is the fact that in marine systems, without continued monitoring, it is often difficult to determine the early (and likely asymptomatic) stages of infection. Thus, it is possible that the commonly reported disease symptoms (e.g., bleaching, spotting, or rotting) for marine organisms are representative of late stages of disease and the proliferation of secondary invaders. Indeed, many features attributed to potential pathogen/s, such as the ability to colonize and degrade host tissue, could also be utilized by saprophytic organism/s, further obscuring the identity of the true primary pathogen/s.

A second explanation is that disease is not the result of a single microbial agent, but rather a consortium of microbial species (i.e., polymicrobial) with each member playing a distinct role in the pathogenic process. The black band disease (BBD) of corals is arguably, to date, the best model for polymicrobial disease in marine systems, with a complex microbial community consisting of cyanobacteria, heterotrophic and sulfur-cycling bacteria, as well as archaeal members implicated in disease progression (Sato et al., 2016).

A third possibility is that some microbial pathogens exist as part of a host’s native microbiome, and under certain conditions, i.e., host senesce, can switch from a commensal to a pathogenic lifestyle. For example, the marine bacterium *Phaeobacter gallaeciensis* BS107 produces antibacterial and growth promoting compounds that are beneficial to its host microalga, *Emiliania huxleyi*; however, upon detection of algal breakdown products, the beneficial bacterium is converted into an opportunistic pathogen through the production of algaecide compounds (Seyedsayamdost et al., 2011). In such cases it can be difficult to apply classical measurements of disease causality, such as Koch’s postulates (see **Table 1** for definition), which assume that the causative agent is always associated with disease while being completely absent in healthy individuals (Koch, 1882; Kaufmann and Schaible, 2005).

**FIGURE 1:**
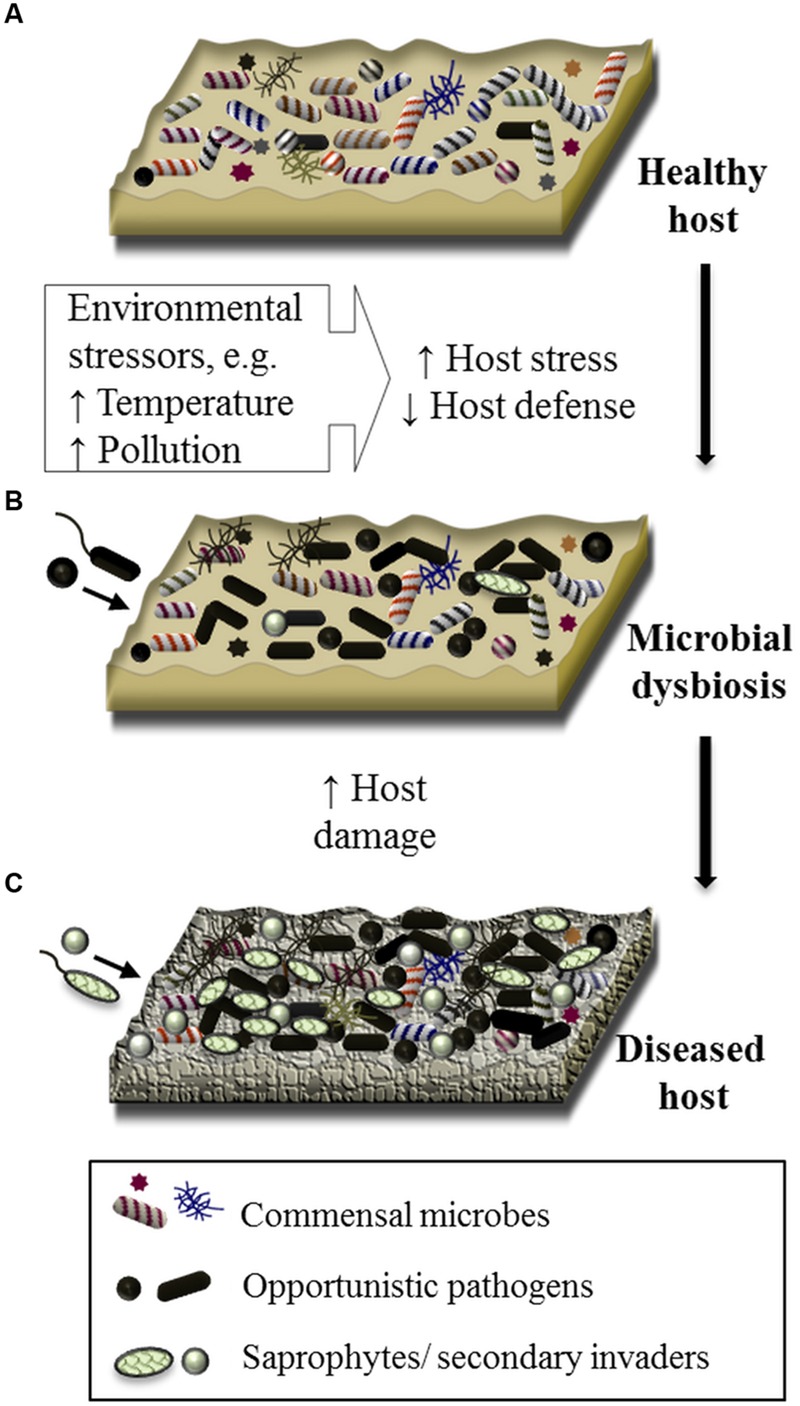
**Schematic of the proposed involvement of microbial dysbiosis, opportunistic pathogens and polymicrobial interactions in the disease of marine organisms.** The surface of a healthy marine living organism (healthy host) is colonized by a consortium of commensal microorganisms, which may include bacteria, archaea, microbial eukaryotes, and viruses **(A)**. Under conditions of elevated environmental pressure, the host can become stressed negatively impacting the host defence mechanisms. On this susceptible host, opportunistic pathogens and/or pathogenic consortia (i.e., polymicrobial infection), originating from the healthy host or from the surrounding environment, proliferate, resulting in a state of microbial dysbiosis **(B)**. Microbial pathogens along with saprophytes or secondary colonizers further cause damage to the host resulting in visible disease **(C)**.

Finally, a forth and often overlooked explanation is that disease results from the proliferation of any number of opportunistic pathogens when there is dysbiosis in the host microbiome as a result of environmental pressures and/or host stress (Lesser et al., 2007; Burge et al., 2013; Egan et al., 2014) (**Figure 1**). Microbial dysbiosis has emerged as an explanation for several acute and chronic human diseases, including atopic dermatitis of the skin (Brüssow, 2015), gastrointestinal disorders (Clemente et al., 2012; Parekh et al., 2015), periodontal disease (Meyle and Chapple, 2015), and inflammatory respiratory diseases (Mahdavinia et al., 2016). In these examples, scientists and medical professionals suggest that a combination of environmental, immune, and host genetic immune factors, together with microbiome disturbance, play a role in the establishment and severity of these diseases (Legatzki et al., 2014; Butto et al., 2015; Donaldson et al., 2016; Hajishengallis and Lamont, 2016). For example, lower microbial diversity and depletion of commensal bacteria in the gut is associated with the development of asthma and Crohn’s Disease, implicating a role for microbial dysbiosis in these now common diseases (Frank et al., 2007; Candela et al., 2012; Abrahamsson et al., 2014). Further, there is a growing appreciation for the complex interplay between beneficial species, opportunistic pathogens, environmental factors and the immune system, and the influence that these interactions have on human health outcomes, including the onset of chronic disease (Mazmanian et al., 2008; Chow and Mazmanian, 2010; Butto et al., 2015).

While less understood, there is evidence for dysbiosis syndromes in marine systems. Meres et al. (2012) proposed microbial dysbiosis and the emergence of opportunistic pathogens (including the previously reported *Aquimarina* spp. pathogens) as a mitigating factor in the epizootic shell disease (ESD) of lobsters. Likewise, analysis of microbial communities associated with healthy and diseased *Montastraea* corals indicated multiple etiological factors, including polymicrobial infections, are likely to be responsible for BBD (Miller and Richardson, 2015) and white-plague disease (Cook et al., 2013). Studies investigating the impact of environmental change on marine hosts have given further insight to the role of microbial dysbiosis and disease. Exposure to stressful environmental conditions results in distinct shifts in microbial community assemblages that can be detected prior to any physical sign of disease in corals (Webster et al., 2013; Closek et al., 2014), seaweeds (Fernandes et al., 2012; Stratil et al., 2013) and sponges (Webster et al., 2008; Fan et al., 2013) (**Figure 1**). In some cases, these shifts have been characterized by an increase in groups of pathogenic bacteria (e.g., *Vibrio* spp. (Tout et al., 2015), Roseobacter species (Zozaya-Valdes et al., 2015)) and/or an increase in the abundance of virulence-related functional genes (Vega Thurber et al., 2009; Littman et al., 2011; Fernandes et al., 2012). This may be considered early evidence for dysbiosis and the enrichment of bacteria with pathogenic potential. Thus, a disease framework centered around stress-induced dysbiosis and the emergence of opportunistic pathogens would not only explain inconsistencies in laboratory infection assays and pathogen detection in characterized diseases (e.g., Rosenberg et al., 2009; Meres et al., 2012; Cook et al., 2013; Joyner et al., 2015; Zozaya-Valdes et al., 2015), but would also help to understand the etiology of yet undefined syndromes.

## A Call for a Fresh Approach to Characterize and Manage Marine Dysbiosis and Disease

Historically, studies of host–pathogen interactions have centered on the detection and control of the infectious agent/s. For decades researchers have studied the detailed molecular mechanisms that enable pathogenic microorganisms to infect their host and cause disease. These studies have resulted in a wealth of knowledge, including the identification of growth and virulence factors [e.g., toxins, enzymes, and host colonization factors (Finlay and Falkow, 1997)]. There is no doubt about the value of such an approach to disease control, especially where an understanding of pathogenesis has facilitated the development of vaccines and antibiotics to treat highly infectious pathogens such as those responsible for measles, diphtheria, and typhoid. However, if dysbiosis, opportunistic pathogens and/ or polymicrobial infections are responsible for disease, a paradigm shift in the way we think about host health and microbial symbiosis is required.

The importance of this paradigm shift has recently been recognized by medical microbiologists with calls to “ditch the term pathogen” (Casadevall and Pirofski, 2014; Altmann and Boyton, 2015) and to place greater emphasis on the complex interplay between the host and its microbiome (Méthot and Alizon, 2014). The importance of a less ‘pathogen-centric’ view of disease is now being recognized (Casadevall and Pirofski, 2015) and recent studies have also questioned the value of using common virulence genes alone as predictors of a bacterium’s ability to cause disease (de Lorenzo, 2015; Hilker et al., 2015; Wassenaar and Gunzer, 2015). Indeed, many virulence traits in well-studied pathogens have secondary roles [or ‘dual function roles’ (see **Table 1** for definition)] in persistence and stress adaptation outside the host environment (Casadevall et al., 2003; Vezzulli et al., 2008). Therefore, the discovery of a high proportion of virulence genes in the genomes of ‘non-pathogenic’ marine bacteria (Thomas et al., 2008; Persson et al., 2009; Gennari et al., 2012) and the dual function of known virulence factors (Erken et al., 2013; Gardiner et al., 2014) further speaks to the need for a broader contextual view of disease in the marine environment.

One of the first challenges in understanding the role of dysbiosis in marine diseases is obtaining suitable baseline data on what is considered a “healthy” microbiome. While the past decade has seen an increase in marine microbiome studies, inherent natural variation, inconsistency in sample collection and different approaches to data analysis means it is difficult to compare data across individual studies. Thus, there is an urgent need for larger spatial and temporal scale microbiome studies, particularly of species that are habitat-forming (e.g., macroalgae, corals), important to food security (e.g., farmed finfish and invertebrate species), or at high risk of disease (e.g., threatened or endangered species). For example, by analyzing the microbial diversity associated with over 250 samples covering a large spatial distribution of the reef forming kelp *Ecklonia radiata*, Marzinelli et al. (2015) identified a consistent microbiome for healthy individuals. However, when significant deviations in the kelp microbiome occurred, they could be correlated with visible signs of disease. Thus, with finer temporal sampling, microbial community data may be an effective environmental monitoring tool to detect microbiome changes as predictors for disease outcomes.

Despite the decreasing cost of characterizing microbial communities via sequencing, the scale required to adequately obtain the baseline data remains prohibitive to most individual research laboratories. Therefore, continued engagement of community and philanthropic supported initiatives will be essential. Such large scale microbiome initiatives have proven to be successful strategies for the analysis of oceanic microorganisms [e.g., GOS (Yooseph et al., 2007) and TARA (Pesant et al., 2015)], the human microbiome^1^ and other environmental systems [(e.g., Earth Microbiome Project^2^ (EMP)], with recent calls to move to an international microbiome initiative (IMI) that encompasses all habitats and is not limited by national boarders (Dubilier et al., 2015). We suggest that such global pattern analysis should be extended to include temporal studies of the typical microbiome of host species to gain insight into the emergence of dysbiosis and disease.

To accurately discover and manage the factors that contribute to dysbiosis and disease in marine systems requires a true holobiont approach (i.e., host and associated microbiome as one entity see **Table 1**). As stated above, such an approach needs to go beyond correlating health with the presence of specific groups of bacteria. Indeed, studies of vibriosis in oysters are finding that diseased animals are often colonized by diverse populations of *Vibrio* strains, rather than single virulent isolates (Lemire et al., 2015). Such findings further highlight the need to move beyond simple characterization of single pathogens and towards an understanding of how the host and its microbiome respond to, and interact with, each other, and how these interactions are influenced by environmental factors. Microbiologists, ecologists, environmental scientists, system biologists, and host physiologists will need to work closely together and occasionally step out of their comfort zone. For example, molecular microbiologists will need to become less reductionist and accept that the answers maybe more complex and variable than they feel confident with. Likewise (macro-) ecologists will need to embrace the concept that the organisms they study are not functioning on their own, but are influenced by millions of microorganisms that are not always simple to measure or control.

Compared to the medical field the understanding of dysbiosis in marine systems is in its infancy. In the last 10 years, over 1020 articles have been published on microbial dysbiosis in humans alone (using keywords “dysbiosis human”, PubMed.gov search 10.02.16). However a similar search on marine organisms (using keywords “marine dysbiosis”) retrieved only three publications. Whilst one can argue that the scientific breakthroughs in the medical field are due to the significantly larger research support (in terms of numbers of scientists and research funding), an important consideration is that this research effort is going towards one model organism, i.e., humans. This is in contrast to work on marine symbiosis, where relatively smaller amounts of money are spread across a large range of host species. Thus, in order for marine symbiosis research to come close to the equivalent depth of understanding of dysbiosis and disease as in the medical field, basic principles may need to first be studied collectively on fewer model species. The choice of model host/s should be made by the science community taking into consideration accessibility, ecological context, available metadata, as well as their ability to grow in aquaria and be experimentally manipulated. Indeed, some excellent marine model organisms exists to study microbial interactions and/or disease in macroalgae [e.g., *Ectocarpus siliculosus* (Zambounis et al., 2013) and *Delisea pulchra* (Harder et al., 2012)], invertebrates [e.g., oysters *Crassostrea gigas* (Le Roux et al., 2016); and cnidarians such as species of *Aiptasia* (Weis et al., 2008) and *Hydra* (Bosch, 2014)], vertebrates (e.g., zebrafish *Danio rerio* (Rawls et al., 2006), and medaka *Oryzias latipes* (Schartl, 2014)), and these systems should be further explored.

In addition it is important to consider how far the current theoretical framework of disease (predominately established for terrestrial systems) can be applied to marine systems. Indeed, McCallum et al. (2004) raised this question over 10 years ago and argued that, while the same theory may be applicable to marine systems, fundamental differences between marine and terrestrial systems may limit its usefulness. For example, marine systems will require their own set of disease models and management strategies that take into account the different life histories of marine and terrestrial organisms, the different modes of dispersal and recruitment of both microorganisms and their hosts (i.e., the “open” nature of marine systems), and differences in the impact of environmental change on marine and terrestrial systems (McCallum et al., 2004; Pollock et al., 2011; Arnold et al., 2012; Garren and Azam, 2012; Wahl et al., 2012; Burge et al., 2014).

## Conclusions and Perspectives

Ever since the early descriptions of the infectious agents by Robert Koch in the 1800s, the study of microbial diseases has been overshadowed by the desire to discover and eliminate the causative pathogen. However, as our understanding of microbial ecology and host–microbial interactions improve it is becoming increasingly clear that the relationship between a host and its microbiome is complex, and the lines between commensal microorganisms and pathogens are often blurred.

Next generation ‘–omic’ technologies are living up to the promise of providing the data resolution and scale required to understand many of these complex interactions. However, major questions remain unanswered, including: How are microbiomes established and maintained? What specific functions do microbiomes play in host resilience, and how do changes in microbial community structure impact host health and disease? Answers to these questions and the need to rethink the definitions of disease are particularly pertinent to marine scientists today in the face of environmental change and increasing observations of disease inflicting marine organisms.

## Author Contributions

SE and MG both made a direct contribution to planning and writing of the manuscript and both have approved it for publication.

## Conflict of Interest Statement

The authors declare that the research was conducted in the absence of any commercial or financial relationships that could be construed as a potential conflict of interest.

## Footnotes

1 http://www.human-microbiome.org/

2 http://www.earthmicrobiome.org/
